# Ecometabolomics of Loggerhead Sea Turtles (*Caretta caretta*): The Impact of Age on Metabolomic Profiles

**DOI:** 10.3390/ijms26020545

**Published:** 2025-01-10

**Authors:** Pablo Jesús Marín-García, Daniel García-Párraga, Jose Luis Crespo-Picazo, Lola Llobat, María Cambra-López, Francesco Bordignon, Juan José Pascual, Torben Larsen, Mette Skou Hedemann

**Affiliations:** 1 Department of Animal Production and Health, Veterinary Public Health and Food Science and Technology (PASAPTA), Facultad de Veterinaria, Universidad Cardenal Herrera-CEU, CEU Universities, 46113 Valencia, Spain; maria.llobatbordes@uchceu.es; 2 Fundación Oceanogràfic de la Comunitat Valenciana, Ciudad de las Artes y las Ciencias, 46013 Valencia, Spain; dgarcia@oceanografic.org (D.G.-P.); jlcrespo@oceanografic.org (J.L.C.-P.); 3 Institute for Animal Science and Technology, Universitat Politècnica de València, 46022 Valencia, Spain; macamlo@upvnet.upv.es (M.C.-L.); jupascu@dca.upv.es (J.J.P.); 4 Department of Agronomy, Food, Natural Resources, Animals and Environment (DAFNAE), University of Padova, Viale dell’Università 16, 35020 Padova, Italy; francesco.bordignon@unipd.it; 5 Department of Animal and Veterinary Sciences, Aarhus University, Blichers Alle 20, DK-8830 Tjele, Denmark; torben.larsen@anivet.au.dk (T.L.); mette.hedemann@anivet.au.dk (M.S.H.)

**Keywords:** ecometabolomics, nutrimetabolomics, nutritional metabolites, lysophospholipid

## Abstract

To investigate the impact of age on the metabolomic profile of loggerhead sea turtles (*Caretta caretta*), this study analyzed 100 plasma samples of individuals across two age groups—50 post-hatchlings and 50 juveniles—from various locations along the Mediterranean coastline. Both targeted and untargeted metabolomic analyses were performed on the samples. Our results demonstrated a significant age-related effect on the metabolomic profiles in both analyses. Specifically, post-hatchling turtles exhibited increased levels of urea (*p* < 0.001), triglyceride (*p* = 0.0003), cholesterol (*p* < 0.001), lysoPE (18:1/0:0) (*p* < 0.001), 7-HDoHE (*p* = 0.0121), pyrrolidinebutanoic acid (*p* < 0.001), formiminoglutamic acid (*p* < 0.001), pyroglutamic acid (*p* < 0.001), lysoPC (0:0/20:4) (*p* < 0.001), lysoPE (22:6/0:0) (*p* < 0.001), 1-acyl-sn-glycero-3-phosphocholine (*p* < 0.001), DL-homocysteine (*p* < 0.001) and gamma-Glutamyltyrosine (*p* < 0.001). Conversely, post-hatchlings showed reduced levels of total protein (*p* < 0.001), glucose (*p* = 0.0002), uric acid (*p* < 0.001), inorganic phosphorus (*p* = 0.0018) and calcium (*p* = 0.0410) compared with juveniles. These findings suggest significant physiological changes between the age groups, likely due to differentiated feeding patterns. Further research is needed to better understand the metabolic profiles and complex physiological and nutritional interactions of this species.

## 1. Introduction

The concept of ecometabolomics, defined as the application of untargeted metabolomics to explore the interactions between living organisms and their environment, has only recently been described [1,2,3,4,5,6]. Nevertheless, an increasing number of studies are employing this approach to address ecological questions. Although the majority of studies have been conducted on plants, there is an increasing number of studies being applied to animals, covering topics as diverse as the venom of certain frogs [7], physiological responses such as the reproductive status of mammals [8] or exposure to pollutants in marine animals [9], among others.

A new approach in nutrition research using untargeted metabolomics as an exploratory tool is known as nutrimetabolomics [10]. The metabolome/nutrition interaction opens new avenues of research from the search for biomarkers of a specific nutrient deficiency/excess to trials aimed at understanding the nutritional requirements of animals [11]. In this context, the use of this tool becomes particularly relevant for the conservation of certain species [12].

The loggerhead sea turtle (*Caretta caretta*) is a generalist species with a global distribution and is one of the most commonly encountered sea turtles in many regions [13,14]. Recently, it has been classified as “vulnerable” on the International Union for Conservation of Nature’s Red List [15]. Several significant threats are likely impacting loggerhead populations worldwide [16]. In this context of vulnerability, studying the nutrition of this species becomes particularly important.

From a dietary perspective, the loggerhead sea turtle is considered to be an omnivorous species [17], with a diet that varies throughout its life stages and is dependent on its habitat [18,19]. Nutrition plays a key role in the success of many animal species [20]. In the case of the loggerhead turtle, aspects such as the relationship between nutrition and health have been explored [21]. Although further research regarding nutrition and adaptative success is needed, some studies have been able to link aspects relevant to adaptive success as a positive correlation between various mineral concentrations provided by the diet and hatching success in *Caretta caretta* nests [22,23,24,25,26,27].

Thus, the primary objective of the present investigation was to determine whether there was a distinct metabolomic profile associated with different physiological stages by comparing post-hatchlings and juveniles. If such differences existed, the aim was to identify the metabolites that varied in abundance between stages. This would contribute to quantifying the species’ nutritional requirements and enhance our understanding of the loggerhead sea turtle’s dietary needs.

## 2. Results

**Figure 1:**
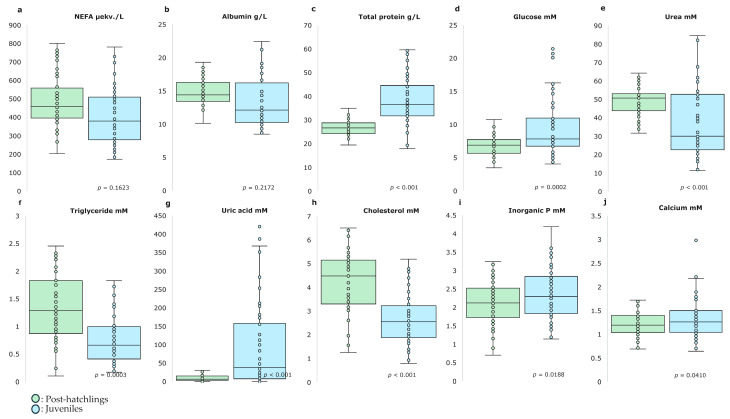
Effect of age on analyzed nutritional metabolites of loggerhead sea turtles (*Caretta caretta*). NEFA: non-esterified fatty acid (n = 100; 50 post-hatchlings and 50 juveniles).

Figure 1 presents a summary of the impact of age on the nutritional metabolites analyzed using targeted metabolomics. A significant effect of age on most of the analyzed metabolites was observed. Although no differences were observed in NEFA and albumin, post-hatchling animals showed higher urea (+35%; *p* < 0.001), triglyceride (+77%; *p* = 0.0003) and cholesterol (+61%; *p* < 0.001) than juvenile animals. Nevertheless, juveniles showed higher total protein (+48%; *p* < 0.001), glucose (+37%; *p* = 0.0002), uric acid (+539%; *p* < 0.001), inorganic P (+12%; *p* = 0.0018) and calcium (+10%; *p* = 0.0410) than post-hatchlings. These results indicate that age significantly modulates the concentration of several key nutritional metabolites, likely reflecting developmental shifts in the metabolic pathways associated with nutrient assimilation, utilization and storage.

**Figure 2:**
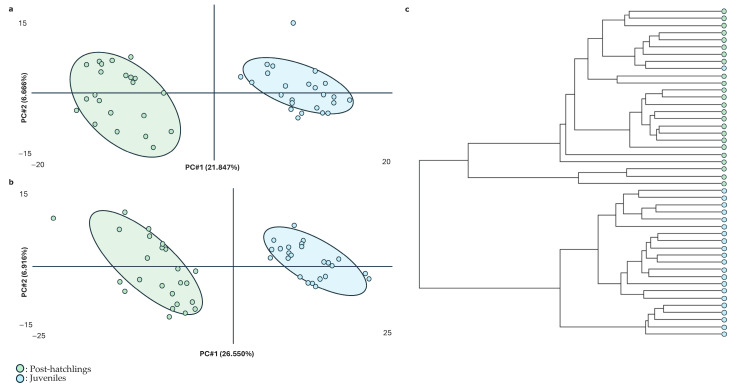
Summary of the results obtained from the untargeted metabolomic testing of loggerhead sea turtles (*Caretta caretta*). (**a**,**b**) PLS models of plasma in negative (R^2^ = 0.9618) and positive (R^2^ = 0.9640) modes, respectively. (**c**) Dendrogram of the different samples obtained from ultra-high-performance liquid chromatography and mass spectrometry in both positive and negative modes (n = 48; 24 post-hatchlings and 24 juveniles). PC#: principal component.

Figure 2 summarizes the results using untargeted metabolomics. Figure 2a,b depict the first two principal components obtained through the PLS-DA of the untargeted metabolomic data in positive and negative modes, respectively. As demonstrated, the variability linked to these principal components extracted from the metabolic profile (on average, 32% of the total) could effectively distinguish between physiological states as there was no overlap between the two ages. Furthermore, the dendrogram illustrated a strong classification of the samples, supporting the separation of metabolic profiles based on age. This indicates that the metabolic signatures of post-hatchling and juvenile animals are distinct and can easily be distinguished using untargeted metabolomics.

**Figure 3:**
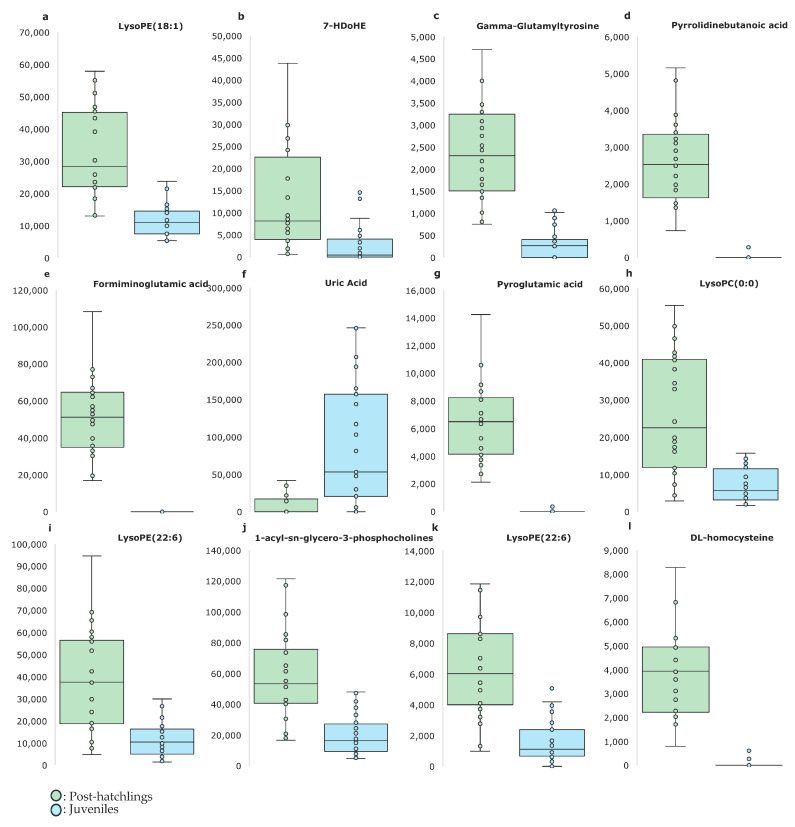
Effect of age on the main discriminant metabolites analyzed using the untargeted metabolomics of loggerhead sea turtles (*Caretta caretta*). (**a**) C_23_H_46_NO_7_P—LysoPE (18:1/0:0), (**b**) C_22_H_32_O_3_—7-HDoHE, (**c**) gamma-Glutamyltyrosine, (**d**) pyrrolidinebutanoic acid, (**e**) formiminoglutamic acid, (**f**) uric acid, (**g**) pyroglutamic acid, (**h**) C_28_H_50_NO_7_P—LysoPC (0:0/20:4), (**i**) C_27_H_44_NO_7_P—LysoPE (22:6/0:0), (**j**) C_26_H_52_NO_7_P—1-acyl-sn-glycero-3-phosphocholine, (**k**) C_27_H_44_NO_7_P—LysoPE (22:6/0:0), (**l**) DL-homocysteine. The units on the *x*-axis correspond with the peak area (n = 48; 24 post-hatchlings and 24 juveniles).

Table 1 provides a summary of the identified metabolites responsible for the observed differences between age groups. A comparison of metabolite intensities is shown in Figure 3. Post-hatchling animals showed higher intensities of lysoPE (18:1/0:0) (+210%; *p* < 0.001), 7-HDoHE (+360%; *p* = 0.0121), pyrrolidinebutanoic acid (+5127%; *p* < 0.001), formiminoglutamic acid (+105%; *p* < 0.001), pyroglutamic acid (+17,201%; *p* < 0.001), lysoPC (0:0/20:4) (+259%; *p* < 0.001), lysoPE (22:6/0:0) (+235%; *p* < 0.001), 1-acyl-sn-glycero-3-phosphocholine (+193%; *p* < 0.001), lysoPE (22:6/0:0) (+289%; *p* < 0.001), DL-homocysteine (+10,860%; *p* < 0.001) and gamma-Glutamyltyrosine (+210%; *p* < 0.001) than the juvenile animals. However, juvenile animals showed higher uric acid intensities (+210%; *p* < 0.001) than post-hatchling animals. These results suggest that the specific metabolites involved in lipid metabolism, amino-acid biosynthesis and purine metabolism are differentially regulated between post-hatchling and juvenile animals, reflecting the metabolic adaptations that accompany growth and maturation.

**Figure 4:**
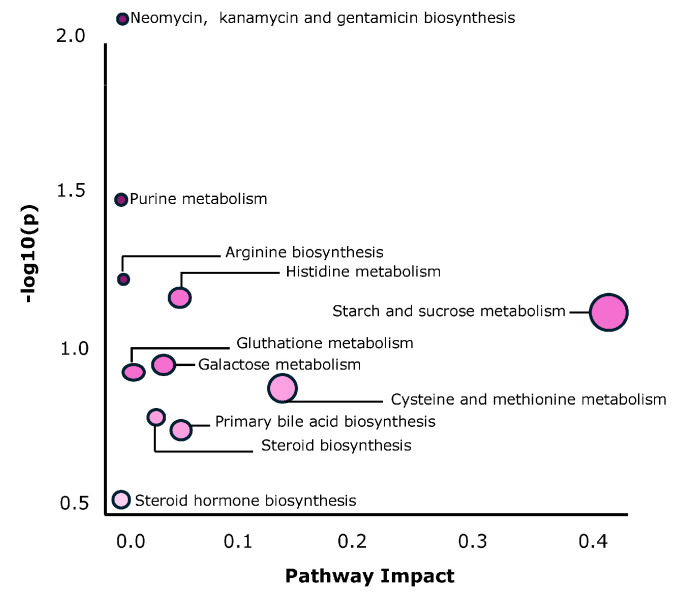
Overview of the pathway analysis plot. A graphical summary of the pathway analysis for loggerhead sea turtles (*Caretta caretta*) combining discriminant metabolites from both targeted and untargeted metabolomics is displayed. In this plot, pathways are depicted as circles, with their color representing the *p*-value and their size reflecting the pathway impact value.

Figure 4 presents the pathway analysis plot, which aligns with the graphical summary of the analysis. In this figure, each matched pathway is depicted as a circle, highlighting neomycin, kanamycin and gentamicin bio-metabolism; starch and sucrose metabolism; and cysteine and methionine metabolism as the most enriched pathways. These findings indicate that the metabolic pathways related to amino-acid and carbohydrate metabolism were particularly prominent in both age groups, with distinct differences in their regulation between post-hatchling and juvenile animals.

In terms of validation, the average R^2^ and Q^2^ values obtained from the PLS analysis were 0.96 and 0.68, respectively.

## 3. Discussion

With the primary aim of determining whether age affects the plasma metabolomic profile of loggerhead sea turtles, an analysis was conducted on 50 post-hatchling individuals and 50 juvenile individuals. As the main tool used was metabolomics, it is necessary to begin by discussing the validation of this technique.

In validating an untargeted metabolomic analysis, R^2^ measures the extent to which the model fits the observed data, while Q^2^ assesses the predictive accuracy. According to the SIMCA user guide, a Q^2^ value greater than 0.5 indicates good predictive performance. Our average results for R^2^ and Q^2^ were 0.96 and 0.68, respectively, suggesting that our models fitted the data well and demonstrated a strong predictive capability. Another aspect to consider was the case of uric acid, which appeared as a major metabolite in juveniles compared with post-hatchlings in both analyses (firstly, when quantitatively analyzed through targeted metabolomics and then as a discriminant metabolite in untargeted metabolomics). In this way, it could be confirmed that both tools (targeted and untargeted metabolomics) used were doubly validated.

Once the validation has been discussed, the differences in the metabolites can then be discussed. As mentioned in the Results section, there was a clearly differentiated metabolomic profile in both the targeted and untargeted analyses. The values of targeted metabolites have previously been studied for *Caretta caretta* and other sea-turtle species, and our values aligned with the ranges reported in most of the reviewed studies [28,29,30,31,32,33,34,35].

The higher levels of total protein, glucose, uric acid, phosphorus and calcium observed in juvenile animals could be explained by increased feeding activity at this stage or high feeding activity. These elevated plasma levels of metabolites could be a result of enhanced catabolic processes (due to excess levels) or greater transport of these metabolites to tissues [36]. Although previous feeding was not measured in this experiment, numerous studies have correlated feeding with animal size [37]. It is important to note that in this study, the juveniles had an average weight of +9.3 kg. A higher level of feeding could explain the elevated metabolic levels, as observed in other studies where certain blood metabolites decreased under dietary restrictions, including in *Chelonia mydas* [38,39]. Therefore, higher levels were expected to be observed in juveniles compared with post-hatchlings.

It is particularly interesting to note that some of the metabolites analyzed using targeted metabolomics (urea, triglyceride and cholesterol) as well as most of the metabolites identified using untargeted metabolomics were found in higher concentrations in post-hatchlings.

Regarding energy metabolism, reduced levels of cholesterol and triglyceride were observed in older animals in our study. These findings were consistent with other studies that reported a decrease in triglyceride levels between 1 and 7 months of age and a reduction in cholesterol levels between 1 and 5 months of age. Continuing with energy metabolism, lysoPE (18:1/0:0), lysoPC (0:0/20:4), lysoPE (22:6/0:0), 1-acyl-sn-glycero-3-phosphocholine and lysoPE (22:6/0:0) belong to the lysophospholipid family; some of them are highly abundant in humans [40,41]. All these metabolites were higher in post-hatchlings than juvenile animals in very similar proportions (on average, 248%). It has been demonstrated that lysophospholipids are involved not only in maintaining normal cell growth but also in the biosynthesis of different metabolites and in promoting rapid growth and reproduction in the body [42,43].

Regarding protein metabolism, higher levels of urea were observed in younger animals in this study. Urea is the final product of protein catabolism. It enters the bloodstream and is subsequently filtered by the kidneys and excreted in the urine of ureolytic animals. Despite being reptiles, the order to which the loggerhead turtle belongs categorizes them as ureolytic animals [44]. Higher urea levels in young animals could be explained either by a higher quantity of diet, a higher total intake of protein or a greater degree of protein turnover. This excessive nitrogen excretion may have potentially negative impacts [45,46]. Gamma-Glutamyltyrosine is classified within the group of N-acyl-alpha amino acids and their derivatives. Pyrrolidinebutanoic acid belongs to the proline family and its derivatives [47], while formiminoglutamic acid is categorized under glutamic acid and its derivatives. Pyroglutamic acid falls within the group of alpha amino acids and their derivatives [48]. Lastly, DL-homocysteine is classified as a standard amino acid. The alteration of all these metabolites suggests that the metabolic pathways related to protein are affected by aging in this species. All these relationships between the differentiated metabolites and the affected metabolic pathways are depicted in Figure 4.

Higher levels of all these metabolites cannot be explained by a greater food intake. There are various hypotheses as to why they are more prevalent in younger animals, including (i) younger animals may proportionally select foods with a higher concentration of certain nutrients; (ii) the observed differences may not be due to the quantity consumed but rather to nutritional utilization efficiency, such as digestibility; (iii) different nutritional requirements could trigger distinct responses at the nutritional level; and (iv) stress from capture may also play a role. Each of these factors is discussed separately below.

Various studies have shown a differentiated selection of specific foods in young turtles, which could result in a more selective intake of certain nutrients. Research indicates that smaller turtles have higher nutrient utilization efficiency, as evidenced by greater digestibility. Digestibility in marine turtles has been studied [49]. In *Pseudemys nelsoni*, it was shown that 12 g hatchlings had significantly better digestive performance compared with 3 kg adults. The results of the present study suggested either a more efficient utilization of nutrients, leading to the observed metabolite levels, or a more selective feeding behavior regarding certain nutrients [50]. Additionally, some metabolites have shown significant differences that may not be linked to the amount consumed (whether in kilograms of feed or protein) or to post-digestion bioavailability but rather to nutritional requirements. For example, in the case of urea, certain essential amino-acid requirements vary depending on the physiological state, which could lead to the presence or absence of limiting amino acids, thereby increasing the levels of this metabolite. Finally, it has been observed that stress related to capture can alter the metabolic profile of certain animals [51,52]. For all these reasons, delving deeper into the nutritional aspects of this species is becoming increasingly necessary.

## 4. Materials and Methods

### 4.1. Animal Ethics Statement

Samples were collected from loggerhead sea turtles (*Caretta caretta*) either reared in a captive management program or obtained from wild individuals incidentally caught by local fisheries or undergoing rehabilitation in Spain. All procedures complied with Spanish regulations. The study was conducted under the authorization of the *Conselleria d’Agricultura*, *Desenvolupament Rural*, *Emergència Climàtica i Transició Ecològica* of the Valencian Community in collaboration with the Fundación Oceanogràfic for marine fauna conservation. Blood sampling was approved by the institution’s Animal Care and Use Committee (Approval No. OCE-5-22). All handling and sampling adhered to veterinary ethical standards and animal welfare guidelines.

### 4.2. Animals and Sampling

A total of 100 loggerhead sea turtles were used in this experiment. Animals were classified according to their physiological status as either post-hatchling (n = 50) or juvenile animals (n = 50). In each experimental group, there were 50% males and 50% females. Following hatching, the turtles were enrolled in a rearing program at the Oceanogràfic Aquarium in Valencia, Spain. Juveniles were obtained during the rehabilitation process of the rescue center at the same institution, originating from different locations in the Mediterranean. This classification was carried out using the known age of individuals from the head-start program established by Casale et al. According to their specific morphological criteria, such as weight (with a mean of 1.3 kg for post-hatchlings and 10.6 kg for juveniles, respectively), straight carapace length (SCL) (with a mean of 17 cm for post-hatchlings and 38 cm for juveniles, respectively), straight carapace width (SCW) (with a mean of 16 cm for post-hatchlings and 32 cm for juveniles, respectively), curve carapace length (CCL) (with a mean of 14 cm for post-hatchlings and 39 cm for juveniles, respectively), the animals were classified either as post-hatchlings or juveniles. All animals underwent a veterinary examination as well as a morphological assessment.

Blood samples were obtained in 2023 during morning hours at the same time of day (approximately 08:00 a.m.). Blood samples were taken from the dorsal cervical sinus using a 1 to 5 mL syringe and a 25 G to 21 G hypodermic needle (Henry Schein^®^, Hong Kong). Blood samples were transferred to 1 mL lithium heparin tubes (Aquisel^®^, Abrera, Spain) for immediate processing. Plasma was separated by centrifugation at 6339× *g* for 5 min and subsequently stored at −80 °C until the analysis. The plasma samples were then subjected to targeted and untargeted metabolomic analyses.

### 4.3. Targeted Metabolomic Analysis

A total of 100 samples (50 for post-hatchlings and 50 for juveniles) was used for the targeted metabolomic analysis. The nutritional metabolites analyzed were non-esterified fatty acid (NEFA), albumin, total protein, glucose, urea, triglyceride, uric acid, cholesterol, inorganic P and calcium.

NEFA was determined using the Wako NEFA C ACS-ACOD assay method. Analyses were performed using an ADVIA 1800^®^ Chemistry System autoanalyzer (Siemens Medical Solutions, Tarrytown, NY 10591, USA).

Albumin, total protein, glucose, urea, triglyceride, uric acid, cholesterol, inorganic P and calcium were determined according to standard procedures (Siemens Diagnostics^®^, Erlangen, Germany; Clinical Methods for ADVIA 1800). For NEFA, triglyceride and uric acid, the intra- and inter-assay CV% variations were below 2.5% and 3.1%, respectively. Similarly, for albumin, creatinine, total protein, glucose, urea, cholesterol, phosphorus and calcium, the CV% variations were consistently below 2.2% and 2.8%.

### 4.4. Untargeted Metabolomic Analysis

In total, 48 plasma samples of randomly selected animals (24 for post-hatchlings and 24 for juveniles) were selected. Further information regarding the untargeted metabolomic analysis, sample quality control, metabolomic data preprocessing and metabolite identification is described in Marín-García et al. (2024) [52]

### 4.5. Statistical Analysis

To examine how the metabolomic profile of loggerhead sea turtles changed with age—specifically, by comparing post-hatchlings with juveniles—two statistical analyses were performed on data from both targeted and untargeted metabolomics.

Metabolites obtained from targeted and untargeted metabolomic analyses were fitted to a normal distribution and analyzed as dependent variables using a GLM model from SAS, V9, including life-stage classes a main fixed effect. Least square means comparisons were performed using a *t*-test.

## 5. Conclusions

The main conclusions drawn from this study are as follows: (i) There was a clear effect of age on plasma metabolites measured using both targeted and untargeted metabolomics. (ii) Post-hatchling turtles had higher levels of urea, triglyceride, cholesterol, lysophospholipids, pyrrolidinebutanoic acid, formiminoglutamic acid, DL-homocysteine and gamma-Glutamyltyrosine as well as lower levels of total protein, glucose, uric acid, inorganic phosphorus and calcium compared with juveniles. (iii) Further studies are needed to explore these complex interactions and improve our understanding of the nutrition of this species.

## Figures and Tables

**Table 1:** List of discriminant plasmatic metabolites between ages (post-hatchlings vs. juveniles) of loggerhead sea turtles (*Caretta caretta*).

m/z-RT	ION	Metabolite	*p*-Value
478.2916RT10.224	[M-H]^−^	C_23_H_46_NO_7_P—LysoPE(18:1/0:0)	<0.001
343.2258RT10.837	[M-H]^−^	C_22_H_32_O_3_—7-HDoHE	0.0121
309.10709RT2.661	[M-H]^−^	Gamma-Glutamyltyrosine	<0.001
229.0813RT1.003	[M-H]^−^	Pyrrolidinebutanoic acid ^1^	<0.001
173.0555RT0.757	[M-H]^−^	Formiminoglutamic acid	<0.001
167.01961RT1.227	[M-H]^−^	Uric acid	<0.001
128.034RT0.752	[M-H]^−^	Pyroglutamic acid	<0.001
568.33643RT9.411	[M+H]^+^	C_28_H_50_NO_7_P—LysoPC(0:0/20:4)	<0.001
526.28967RT9.367	[M+H]^+^	C_27_H_44_NO_7_P—LysoPE(22:6/0:0)	<0.001
522.35211RT10.295	[M+H]^+^	C_26_H_52_NO_7_P—1-acyl-sn-glycero-3-phosphocholine	<0.001
500.27371RT8.816	[M+H]^+^	C_27_H_44_NO_7_P—LysoPE(22:6/0:0)	<0.001
291.04099RT0.828	[M+Na]^+^	DL-homocysteine	<0.001

Tentative identification was performed using MS/MS and METLIN/HMDB databases. ^1^ (2S,3′S)-alpha-amino-2-carboxy-5-oxo-1-pyrrolidinebutanoic acid.

## Data Availability

Pablo Jesús Marín García should be contacted to request data from this study.
